# Reply to Sarıoğlu et al. Comment on “Li et al. Association of Triglyceride–Glucose Index and Coronary Chronic Total Occlusion in Patients Undergoing Coronary Angiography: A Retrospective Study. *J. Cardiovasc. Dev. Dis.* 2026, *13*, 275”

**DOI:** 10.3390/jcdd13080352

**Published:** 2026-07-28

**Authors:** Yan Li, Puhan Song, Mengyi Zheng, Yanyao Jia, Juan Wang, Qian Zhang, Xiaorong Xu, Zhiyong Zhang, Zongsheng Guo, Lin Zhao, Jing Cheng

**Affiliations:** 1 Department of Cardiology, Beijing Chaoyang Hospital, Capital Medical University, Beijing 100020, China; ly18811125001@163.com (Y.L.); 18331341285@163.com (M.Z.); jiayanyao@126.com (Y.J.); wangjuan.goal@163.com (J.W.); zqian604@163.com (Q.Z.); medicinexxr@163.com (X.X.); auysun@126.com (Z.Z.); guozongsheng@sohu.com (Z.G.); 2 Department of Pulmonary and Critical Care Medicine, Xuanwu Hospital, Capital Medical University, Beijing 100053, China; sph98176@163.com

We thank Sarıoğlu et al. [1] for their thoughtful comments on our article [2] and for highlighting several important issues regarding the interpretation of the triglyceride–glucose (TyG) index in patients with coronary chronic total occlusion (CTO). We appreciate their interest in our work and agree that the association between the TyG index and CTO should be interpreted cautiously, particularly in relation to nonlinear risk association, treatment-related confounding, clinical discrimination, heterogeneity of the non-CTO group, and the temporal nature of CTO development.

The nonlinear association observed in our study should be interpreted cautiously. In our study, restricted cubic spline analysis suggested a nonlinear association between the TyG index and the presence of CTO. We interpreted this finding cautiously and discussed how differences in study population, geographic background, cardiometabolic phenotype, lipid-lowering therapy, and model specification may contribute to differences in the observed shape of association. We did not intend to imply a definitive biological threshold. Rather, the nonlinear association should be viewed as hypothesis-generating and requiring confirmation in prospective cohorts.

Medication use represents another important issue when interpreting the TyG–CTO association. Because the TyG index is calculated from fasting triglyceride and fasting glucose levels, lipid-lowering and glucose-lowering therapies may directly influence its value. In our retrospective database, detailed information on statins, fibrates, ezetimibe, PCSK9 inhibitors, insulin, metformin, sodium–glucose cotransporter 2 inhibitors, glucagon-like peptide-1 receptor agonists, and other relevant medications was not systematically available. Therefore, these treatments could not be included in the baseline table or adjusted for in the multivariable models. This limitation was explicitly acknowledged in the published article. We agree that the lack of medication data is an important limitation and may have introduced residual confounding; it may affect the interpretation of TyG as an independent metabolic marker in CTO. Future studies should include detailed medication exposure, treatment intensity, treatment duration, and achieved lipid and glycemic control.

Although detailed medication data were unavailable, several related clinical and biochemical factors were considered in the original analysis. Lipid parameters, including LDL-C and HDL-C, were included in the multivariable model, and diabetes mellitus was further adjusted for in a sensitivity analysis. In addition, patients with a history of percutaneous coronary intervention or coronary artery bypass grafting were excluded to reduce heterogeneity related to prior revascularization. These measures may have partially reduced confounding related to baseline metabolic and cardiovascular status. However, they cannot replace direct information on medication exposure, treatment intensity, treatment duration, or achieved lipid and glycemic control. Future studies should collect these data prospectively. As explicitly acknowledged in the Limitations section of our published article, medication information, including lipid-lowering and glucose-lowering therapies, was not systematically available, and this may have introduced residual confounding.

The limited discriminatory performance of the TyG index should also be clearly distinguished from its statistical association with CTO. In our study, the area under the receiver operating characteristic curve was 0.556, indicating weak discrimination. Therefore, the TyG index should not be used alone to identify or diagnose CTO. The purpose of our study was not to develop a diagnostic model, but to evaluate whether the TyG index was statistically associated with the presence of CTO after adjustment for relevant clinical covariates. Thus, the independent association observed in logistic regression should be distinguished from clinical discrimination or diagnostic utility. At the same time, we believe that the association remains clinically and epidemiologically relevant. The TyG index is easily calculated from fasting triglyceride and glucose levels, which are routinely measured in patients undergoing coronary evaluation. Although it is not a diagnostic marker for CTO, it may provide additional information for metabolic risk characterization and epidemiological profiling of patients with coronary artery disease.

The interpretation of the TyG index in relation to CTO should further consider the heterogeneity of the non-CTO comparator group and the temporal nature of CTO development. Patients without CTO may range from those with normal or non-obstructive coronary arteries to those with severe multivessel coronary artery disease without total occlusion. Therefore, our study cannot determine whether the TyG index is specifically related to CTO formation or more broadly reflects diffuse coronary atherosclerotic burden. In addition, CTO develops over months or years, whereas the TyG index in our study was derived from a single fasting blood sample obtained at the time of coronary angiography. This temporal ambiguity limits causal inference. As also stated in the published article, the cross-sectional design, the single TyG measurement at the time of coronary angiography, and the heterogeneity of the non-CTO comparator group limit causal and CTO-specific interpretation of our findings.

In summary, we agree with Sarıoğlu et al. that our findings should be interpreted as evidence of an association rather than proof of causality, diagnostic value, or a CTO-specific biological mechanism. The main contribution of our study was to externally validate the association between the TyG index and CTO in an independent angiography-based cohort and to show that this association persisted after further adjustment for diabetes mellitus. However, future multicenter longitudinal studies with repeated TyG measurements, detailed lipid-lowering and glucose-lowering medication data, and more homogeneous angiographic comparator groups are needed to clarify whether the TyG index reflects CTO-specific pathobiology or a broader metabolic burden of coronary atherosclerosis.

## Data Availability

The data supporting the findings of this study are not publicly available due to patient privacy and ethical restrictions. The dataset contains sensitive clinical information derived from hospital records and coronary angiography, and public sharing was not included in the ethics approval framework for this study. De-identified data can be made available from the corresponding author upon reasonable request, subject to institutional approval and applicable ethical requirements.
